# Erasing gametes to write blastocysts: metabolism as the new player in epigenetic reprogramming

**DOI:** 10.1590/1984-3143-AR2020-0015

**Published:** 2020-08-04

**Authors:** Marcella Pecora Milazzotto, Camila Bruna de Lima, Aldcejam Martins da Fonseca, Erika Cristina dos Santos, Jessica Ispada

**Affiliations:** 1 Laboratório de Epigenética e Metabolismo Embrionário, Centro de Ciências Naturais e Humanas, Universidade Federal do ABC, Santo André, SP, Brasil; 2 Instituto de Ciências Biomédicas, Universidade de São Paulo, São Paulo, SP, Brasil; 3 Département des Sciences Animales, Centre de Recherche en Reproduction, Développement et Santé Intergénérationnelle, Faculté des Sciences de l’Agriculture et de l’Alimentation, Université Laval, Quebec, Canada

**Keywords:** embryo, metabolism, epigenetic, metaboloepigenetic

## Abstract

Understanding preimplantation embryonic development is crucial for the improvement of assisted reproductive technologies and animal production. To achieve this goal, it is important to consider that gametes and embryos are highly susceptible to environmental changes. Beyond the metabolic adaptation, the dynamic status imposed during follicular growth and early embryogenesis may create marks that will guide the molecular regulation during prenatal development, and consequently impact the offspring phenotype. In this context, metaboloepigenetics has gained attention, as it investigates the crosstalk between metabolism and molecular control, i.e., how substrates generated by metabolic pathways may also act as players of epigenetic modifications. In this review, we present the main metabolic and epigenetic events of pre-implantation development, and how these systems connect to open possibilities for targeted manipulation of reproductive technologies and animal production systems.

## Introduction

The pre-implantation embryo must drive a set of organized events since the earliest stages of development to ensure the generation of totipotent and, subsequently, pluripotent blastomeres that will establish initial cellular lineages. These events include morphophysiological, metabolic and molecular regulation that will lead to pro-nuclei formation, activation of the embryonic genome, cell differentiation, morulae compaction and blastocoel formation. The decisions for each of those events are taken in a dynamic environment and lead to broad-spectrum consequences to embryo metabolism, molecular control, epigenetic reprogramming and developmental capacity. With the growing knowledge of the embryonic response to the environment in several cellular aspects (including *in vivo* and *in vitro* models), the biggest challenge is to unravel the delicate relationship that allows the embryos to modulate the molecular machinery using metabolic tools. In this sense, the term metaboloepigenetics (Donohoe and Bultman, 2012) that defines the relationship between energy metabolism and epigenetic and molecular control, has gained space in studies based on stem cells and embryonic development.

Epigenetics is defined as heritable modifications in nucleic acids and associated proteins that do not involve changes in DNA sequence but might impact the modulation of gene expression. It means that in addition to the primary DNA sequence, much of the information on when and where to start transcription is stored in the form of covalent modifications of DNA, RNAs and chromatin associated proteins. More than 100 covalent modifications have already been identified (reviewed by Mach, 2018 and Michalak et al., 2019). Among them, the methylation and hydroxymethylation of cytosine in DNA, and the acetylation, phosphorylation, SUMOylation or ubiquitination of histones residues [specially lysine (K) and / or arginine (R)] are the most studied, precisely because they are related to the accessibility of the genome to the transcriptional machinery. Epigenetic mechanisms are also controlled by non-coding RNA molecules which are not translated into proteins but exert a significant role in the control of gene expression. These include short-chain nc-RNAs (siRNA, miRNA and piRNAs) as well as long non-coding RNAs (lnc-RNAs) (reviewed by Riddle, 2014).

The concept that the metabolism of a cell is integrated in the regulation of epigenetics and transcription is reinforced by the ability of cells to adapt their metabolic and molecular status in response to extracellular environment and nutrient availability (reviewed by Vander Heiden et al., 2009). Since the metabolites are substrates used to generate chromatin modifications, there is an intriguing but rather complex mechanism that connects energy metabolism and epigenetics. Several enzymes have already been characterized as responsible for inserting or removing epigenetic modifications. The activity of these enzymes is regulated, at least in part, by the presence and quantity of energy substrates (Lu and Thompson, 2012).

In this review, we present a brief statement of changes in mammalian pre-implantation metabolism highlighting how the embryos take stage-specific decisions and how these are critical to successfully initiate the developmental program. We also describe how epigenetic reprogramming can act as controllers of development and cellular fate. We follow presenting the communication between these two cellular events, the metaboloepigenetics. At the end, we conclude with the current limitations encountered in the *in vitro* production system, its consequences to the offspring, and future directions to improve embryo quality and viability.

### The metabolism of the pre-implantation embryo – all in good time

Mammalian embryonic cells present a very characteristic metabolism, slow during the first cleavages, followed by an acceleration to support intense cell proliferation and differentiation over the next stages (Leese et al., 2007). During the first cell divisions, gene transcription is diminished, and the initial metabolism is mainly sustained by transcripts and proteins that were produced and stored during oocyte maturation in several species (Zhang and Smith, 2015).

This quiescence phase is followed by the major activation of the embryonic genome that, in the bovine embryo, occurs between 8 and 16 cells stage and marks a turning point for the developing embryo (Graf et al., 2014). The initiation of transcription requires a lot from the cellular machinery that is responsible for controlling the compaction of the morulae and the development of the blastocyst (Hamatani et al., 2006).

**Figure 1:**
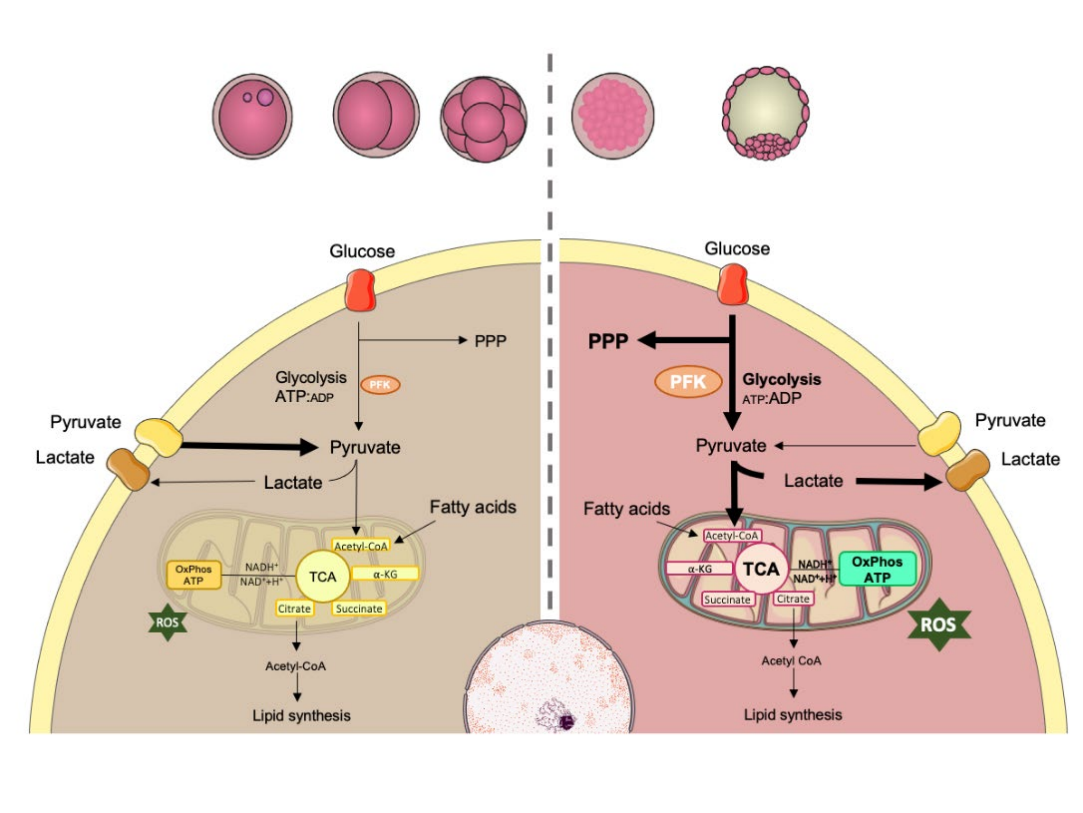
Embryo development requires the activation of specific pathways to produce energy. Prior to compactation, embryo metabolism is mainly supported by pyruvate and amino acids, metabolized through tricarboxylic acid (TCA) cycle and oxidative phosphorylation. At the time of compactation, embryo increases its energetic demand especially for increasing biosynthesis and cell proliferation, but also for the formation and expansion of blastocoel, and hatching. At this time, glucose is metabolized with greater efficiency by two main pathways: the pentose phosphate pathway (PPP) (important for biomass and nucleotide generation) and the glycolytic pathway (that increases ATP production and pyruvate synthesis). At the end of the glycolytic pathway, pyruvate can also be converted to lactate, even in the presence of oxygen. This process is called “aerobic glycolysis” or “Warburg effect”. PFK-phosphofructokinase; ATP-adenosine triphosphate; ADP-adenosine diphosphate; ROS-reactive oxygen species. Adapted from SMART Servier Medical Art image bank (SMART, 2020).

The success of embryo development is dictated by its ability to activate specific energy production pathways (Figure 1). Differential concentrations of glucose, pyruvate, lactate and amino acids are observed in the fluids of bovine oviduct and uterus, reinforcing the idea that pre-implantation embryos need different energy substrates according to their stage of development (Hugentobler et al., 2007; Hugentobler et al., 2008). Considering that, the supplementation of culture media with these fluids has been proposed to improve bovine embryo metabolism and viability (Lopera et al., 2015; Hamdi et al., 2018).

During the first cleavages, bovine embryos, as other mammalian embryos, use pyruvate as the main substrate for energy generation as they have a limited ability to metabolize glucose for this purpose (Guerif et al., 2013). The high ATP: ADP ratio allosterically inhibits the enzyme phosphofructokinase (PKF), the key enzyme in glycolysis, decreasing its affinity to fructose-6-phosphate and limiting the glycolytic pathway, as described in mouse embryos (Barbehenn et al., 1974). At this point, oxidative metabolism and oxygen consumption are also low, probably as a consequence of the quiescent state of the bovine oocyte, reinforcing the importance of oocyte quality and the follicular environment to ensure the proper embryo development (Alves et al., 2019). Amino acids such as glutamine and aspartate are also used for energy generation at this stage via the malate-aspartate transport pathway (MAS) (Lane and Gardner, 2005).

At the time of major embryonic genome activation, the embryo requires more energy to increase biosynthesis and cell proliferation, and also to support the formation and expansion of the blastocoel as well as embryo hatching. This higher energy demand modifies the ATP:ADP ratio, allowing glucose to be metabolized more efficiently, as reported for human and bovine embryos (Devreker, 2007; Guerif et al., 2013). After internalization to the cytoplasm, glucose can follow two main pathways: the pentose phosphate pathway (PPP) or the glycolytic pathway.

In the PPP, ribose chains are generated and later used in the synthesis of DNA and RNA. In addition, the restitution of NADPH from NAD^+^ is required for the reduction of intracellular glutathione, an important antioxidant for the embryos (Wales and Du, 1993; Stincone et al., 2015). Glutathione reduces the levels of intracellular reactive oxygen species, that are generated as a byproduct of the tricarboxylic acid (TCA) cycle and oxidative phosphorylation (Burton et al., 2003). In this context, directing glucose to the PPP can be beneficial for the embryo, as it will inhibit the overflow of substrates into the TCA cycle, thus creating a more suitable redox state for the cells (Harvey et al., 2002).

As previously mentioned, the ATP:ADP ratio is a limiting factor in the glycolytic pathway. Therefore, a reduction in this ratio leads to the activation of PKF and consequently to an increase in the levels of aerobic glycolysis. The pyruvate that is generated through this process is transported to the mitochondria where it is converted to acetyl-CoA by the pyruvate dehydrogenase complex (PDC), thereby connecting glycolysis, which occurs in the cytoplasm, to the tricarboxylic acid cycle (TCA) that occurs inside of the mitochondria. The first reaction in the TCA cycle consists of the transfer of an acetyl group from acetyl-CoA to oxaloacetate, originating the six-carbon compound citrate, which is converted to isocitrate. After oxidation and decarboxylation, isocitrate releases CO_2_ and forms α-ketoglutarate, which also loses CO_2_ and forms succinyl-CoA. The coenzyme is then released producing succinate, which is oxidized to fumarate, which in turn undergoes hydration to form malate. Malate is oxidized to form oxaloacetate and subsequently citrate (Berg et al., 2002). Citrate can also leave mitochondria and be converted to acetyl-CoA in the cytoplasm by ATP citrate lyase. This enzyme is key to the connection of carbohydrate metabolism and lipid metabolism, since the latter requires acetyl-CoA for synthesis (Zaidi et al., 2012).

In the TCA cycle, one molecule of acetyl-CoA is oxidized releasing two molecules of CO_2_, three molecules of NADH, one of FADH2, producing one ATP. NADH and FAD^+^ are metabolic coenzymes that play a critical role in the generation of ATP through oxidative phosphorylation. Within mitochondria, oxidation occurs from NADH to NAD^+^ and FADH2 to FAD^+^ in complexes I and II of the electron transport chain, which leads to the donation of electrons to molecular oxygen. The redox ratio (FAD^+^ / NADH) can be a measure of the cells redox state and has been used *in vitro* and *in vivo* to track metabolic changes during cell differentiation and malignant transformation (Yanes et al., 2010). Changes in a cells redox state can be interpreted as a relative change in the rate of glucose catabolism to oxidative phosphorylation as well.

At the end of the glycolytic pathway, the enzyme lactate dehydrogenase (LDH) may also promote the conversion of pyruvate into lactate, even in the presence of oxygen. This process is called “aerobic glycolysis” or “Warburg effect” and is mainly observed in highly proliferating cells, such as tumoral cells, which to a certain level, are metabolically similar to embryonic cells (Warburg et al., 1927 and reviewed by Krisher and Prather, 2012). Lactate produced by the blastocyst through this pathway may play an important role in key events related to the implantation process, facilitating invasion, proliferation, angiogenesis and modulation of the immune response at the site of implantation (proposed by Gardner, 2015).

Lipids are another important substrate for energy production in mammalian oocytes and embryos. Despite the fact that after fertilization the lipid density is barely altered, intracellular lipids can be considered as a potential and more economical source of energy through ß-oxidation, altering mitochondrial activity and ATP production (Sturmey et al., 2009; Dunning et al., 2010; Dunning et al., 2014).

With so many different cards to play, embryos are known for their so-called plasticity, i.e., their high capacity to adapt when facing environmental stress and changes in the availability of substrates. But not surprisingly, this adaptation has a cost and often modifications in the culture system can compromise metabolic and molecular processes, leading to altered viability.

Metabolism is certainly an indicator of viability; however, many questions remain unanswered, such as: what are the impacts of metabolic changes on the molecular and epigenetic control of cells? Are these impacts reversible?

### Epigenetic reprogramming in pre-implantation embryos – holding the cards

**Figure 2:**
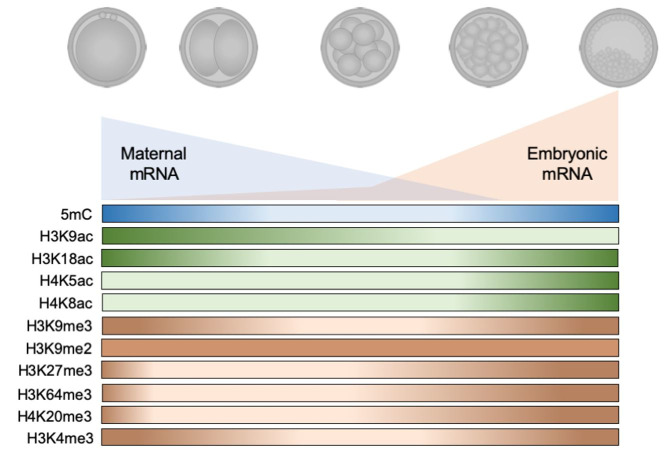
During the pre-implantation development of bovine embryos, the paternal and maternal genome are reprogrammed after fertilization. During this period, the maternal stock of mRNAs is consumed until the embryo is capable of producing its set of transcripts, a critical step known as the major embryonic genome activation. Up to this point, most of the epigenetic marks that were present during the first cleavages were substantially erased (5mC, H3K9ac, H3K18ac, H3K9me3, H3K27me3, H3K64me3, H4K20me3 and H3K4me3). After the major embryonic genome activation all epigenetic marks described for bovine embryos increase, even H4K5ac and H4K8ac that were observed in lower levels since zygote stage. The only exception is H3K9me2 that presents a unique pattern during throughout development. This reprogramming is how the developing embryo ‘writes’ its own profile of epigenetic marks.

Each event during initial embryo development requires changes in metabolism and molecular control and it includes reprogramming of epigenetic settings. This reprogramming occurs to ensure that gametes (highly repressive marks) generate totipotent blastomeres (highly permissive marks) and, subsequently, pluripotent blastomeres of internal cell mass and trophectoderm (more specific marks). The molecular basis for this modification is not yet fully understood but the main events are described below (Figure 2).

### DNA methylation and demethylation

DNA methylation is a chemical alteration promoted by the addition of a methyl group (CH_3_) to the cytosines of DNA molecules (Wu and Zhang, 2014), leading to the formation of 5-methylcytosines (5mC). This modification occurs more frequently in CpG dinucleotides in genomic areas, also known as CpG islands (regions longer than 200 base pairs containing more than 50% CpG dinucleotides), that are located mainly in gene promoter regions (Ioshikhes and Zhang, 2000). In general, DNA methylations are associated with transcription repression, genomic imprinting and post-translational histone modifications (Ioshikhes and Zhang, 2000; Fuks, 2005). In genomic imprinting, one of the gene alleles is silenced by the presence of 5mC, depending on the origin of this allele (maternal or paternal imprinting may be observed, depending on the gene and species) (Barlow and Bartolomei, 2014).

The presence of a methyl group can strongly suppress gene transcription by steric hindrance, inhibiting transcription factors binding and increasing the affinity of Methyl Binding Proteins (MBP) to these gene regions (Lazarovici et al., 2013; Dantas Machado et al., 2015). The MBP are proteins that act to make the access of transcription factors more difficult and can, additionally, lead to the activation of other epigenetic mechanisms, such as histone methylation and deacetylation (Cheng, 2014). However, the effectiveness of DNA methylation in blocking the transcription also depends on the amount, region and size of the region where these methylations are present (Messerschmidt et al., 2014). Generally, high methylation level in promoter regions, especially those with intermediate or high density of CpG dinucleotides, is associated with transcription repression and, therefore, gene silencing (Dupont et al., 2009; González-Recio, 2012; Messerschmidt et al., 2014). On the other hand, promoter regions with low density of CpGs remain transcriptionally active even when hypermethylated (Kulis et al., 2013; Messerschmidt et al., 2014).

The enzymes that catalyze the transfer of a methyl group (CH_3_) from S-adenosylmethionine (SAM) to DNA are called DNA methyltransferases (DNMTs). The DNMTs are responsible for maintaining methylations during DNA replication (DNMT1); inserting methylations into DNA regions without the presence of prior 5mC (DNMT3a and DNMT3b); and regulating DNMT3a or DNMT3b activity (DNMT3l) (Neri et al., 2013; Wu and Zhang, 2014; Messerschmidt et al., 2014; Hervouet et al., 2018). The addition of methylations in DNA without the presence of previous marks, promoted by DNMT3A and DNMT3B, is driven by the interaction between them and some transcription factors that guide methyl groups to specific sites of the genome (Pacaud et al., 2014).

The removal of DNA methyl group, or demethylation, may occur passively or actively and allows epigenetic marks to be erased during development or in response to environmental factors (Messerschmidt et al., 2014; Urrego et al., 2014). Passive demethylation occurs when DNMTs are absent or reduced during DNA replication, which results in the synthesis of a new strand of DNA without methylation and, ultimately, leading to dilution of 5mC (Messerschmidt et al., 2014; Urrego et al., 2014). The active demethylation is initially promoted by enzymes capable to oxidize 5-methylcytosine (5mC) to 5-hydroxymethylcytosine (5hC), which can be converted to 5-formylcytosine (5fC) and 5-carboxycytosine (5caC). These enzymes are known as Ten-eleven-translocation (TET 1-3). The 5fC or 5caC, products of TET activity, are targets of DNA thymidine glycosylase (TDG) and can be processed by the base excision repair (BER) mechanism. Another mechanism responsible for the active DNA demethylation is started by the activation-induced deamination (AID) or apolipoprotein B mRNA catalytic polypeptide (APOBEC1), which converts 5mC and 5hmC into thymidine and 5-hydroxymethyluracil, respectively, leading to the activation of BER. BER mechanism results in the addiction of an unmethylated cytosine to the specific site where the 5mC was present before the beginning of the demethylation process (Delatte et al., 2014; Messerschmidt et al., 2014).

The DNA methylation profile in somatic cells is mostly stable and hereditary during replication. Nevertheless, during gametogenesis and initial embryonic development, the genome of germ cells is epigenetically reprogrammed. This reprogramming consists in erasing 5mC by demethylation processes (either passive or active) and add ‘de novo’ methylations (addition of 5mC in new and specific regions of the genome) among other epigenetic modifications such as histone post-translational alterations. During embryonic development, this reprogramming is initially driven by the stock of maternal DNMTs and TETs present in the oocyte and later, by the expression of specific enzymes after the major embryonic genome activation (Messerschmidt et al., 2014; Atlasi and Stunnenberg, 2017).

### DNA methylation/demethylation in bovine embryos

Mammalian gametes have two different waves of reprogramming, the first one occurring during gametogenesis and the second one during the first days of embryonic development (Haaf, 2006; Seisenberger et al., 2012; Saadeh and Schulz, 2014). The reprogramming cycle of primordial germ cells is barely described in bovine, however, in mouse, it is known that the genome of primordial germ cells is demethylated and a new profile is established with specific patterns for male or female gametes (Seisenberger et al., 2012; Saadeh and Schulz, 2014). The resulting sperm cells and oocytes have higher levels of DNA methylations than somatic cells and, for bovines, male gametes present even greater number of 5mC than the female ones (Zhang et al., 2016; Duan et al., 2019).

After fertilization of gametes, a second cycle of demethylation begins and the highly methylated paternal and maternal genomes are actively and passively demethylated, except for some genes and regions (approximately 100-200 genes and some retrotransposons) (Zhang et al., 2016). Demethylation at this point is mandatory, since the highly methylated genome from gametes must be erased in order to produce totipotent blastomeres, capable of being responsive to specific marks for cell differentiation. The embryonic genome is actively demethylated during the first cleavages through the enzymatic activity of the TETs, and passively demethylated due to the low presence of DNMT1 enzyme (Messerschmidt et al., 2014; Urrego et al., 2014). In the absence of DNMT1, the DNMT3a and DNMT3b enzymes act to guarantee the maintenance of some DNA methylations, preserving the imprinting of some genes (Okano et al., 1999). This demethylation process occurs until a large number of 5mC were removed and the embryos reaches its lowest level of DNA methylation. After that, ‘de novo’ methylation begins to result in the formation of a blastocyst with its own particular profile of DNA methylation e.g. the DNA methylation profiles stabilized for POU5F1, SOX2, NANOG and CDX2 (Urrego et al., 2014; Zhang et al., 2016).

In bovine, the timing of the ‘de novo’ methylation is not consensus. Some reports demonstrated that the ‘de novo’ methylation starts after the embryo reaches the 8-16 cells stage, while others verified a later activation in the remethylation process (Dean et al., 2001; Hou et al., 2007; Dobbs et al., 2013; Zhang et al., 2016). This variation can either be species-specific or a consequence of non-optimized culture conditions. For bovines, we even have variations in DNA methylation profiles due to different sub-species or crossbreed animals used as model (Dobbs et al., 2013; Salilew-Wondim et al., 2015; Urrego et al., 2017), although there is no report in literature directly comparing its variations between *Bos taurus* versus *Bos indicus*. However, it is important to highlight that, even with variations, the process of demethylation followed by remethylation was observed in all studies. Furthermore, ‘de novo’ DNA methylation seems to occur differently between blastomeres, and there are also controversial reports whether the cells with higher levels of DNA methylation are in the inner cell mass or in the trophectoderm (Dean et al., 2001; Hou et al., 2007; Dobbs et al., 2013).

These variations in DNA methylation levels are necessary during embryonic development and can be affected by environmental factors. The use of assisted reproductive techniques, such as superovulation protocols, *in vitro* production (IVP) of embryos and cryopreservation, may affect the DNA methylation pattern in gametes, embryos and offspring. In cattle, the use of assisted reproductive techniques can result in epigenetic reprogramming failures and incapacity to maintain parental imprinting, resulting in problematic phenotypes such as the Large Offspring Syndrome. During bovine embryonic development *in vitro*, some genes that must remain imprinted in one of the alleles are abnormally demethylated (Chen et al., 2017). The longer the *in vitro* culture period of these embryos is, higher is the degree of DNA methylation deregulation in promoters and other gene regions. In addition, if blastocysts are produced using entirely *in vitro* process, they present increased levels of DNA methylation than their *in vivo* counterparts, particularly in gene and promoter regions (Salilew-Wondim et al., 2015).

### Mitochondrial DNA methylation/demethylation

Intense investigation of epigenetic mechanisms is still shaping our understanding on the dynamics that regulates nuclear genome. Direct or indirectly, mitochondria activity has been implicated in these events because it controls numerous epigenetic enzymes. However, the existence of an autonomous mitochondrial epigenetic regulation has been the subject of a long debate over the years with controversial reports stating the presence or absence of mtDNA methylation (Patil et al., 2019). With the improvement of detection techniques, recent studies proposed that similarly to nuclear DNA, mtDNA is also subjected to epigenetic modifications that can influence mitochondrial biogenesis, gene expression and function (Zhang et al., 2019a). A recent study demonstrated non-random patterns of mtDNA methylation, predominantly in a non-CpG context when comparing normal versus liver and breast cancer cells (Patil et al., 2019). In mice, mtDNA methylation profiles also show dynamic local and global changes during development and aging (Dou et al., 2019).

In the bovine model, previous data demonstrated that mtDNA methylation shows particular signatures between oocytes obtained from distinct follicular environments (OPU vs. abattoir ovaries); those signatures are also reflected in the blastocysts produced with these oocytes (Sirard, 2019). Interestingly, the mtDNA methylation negatively correlates with mitochondrial gene expression profile, which may have important consequences to mitochondrial function during preimplantation development. Finally, in the porcine model, mtDNA sequences, including the D- loop control region, have been found to be hypermethylated in oocytes obtained from gilts with polycystic ovaries, contributing substantially to mitochondrial malfunction and decreased oocyte quality (Jia et al., 2016).

Recently, an isoform of DNMT1 was identified to target mitochondria and methylate mtDNA influencing mitochondrial activity (Saini et al., 2017). More specifically, there is evidence showing an upregulation of DNMT1 combined with hypermethylation of mtDNA and altered gene expression in porcine oocytes. However, more evidences are necessary to confirm such connection and to understand the full potential of this type of epigenetic control in the phenotype of the cells.

### Post-translational histone modifications

Histones, composed of a globular C-terminal domain and a flexible N-terminal tail, are the basic components of the nucleosomes, which in turn constitute the chromatin. Each nucleosome consists of a histone octamer containing pairs of each histone (H2A, H2B, H3 and H4) surrounded by 146-147 base pairs of DNA (Luger et al., 1997; Ettig et al., 2011). The N-terminal tail, which protrudes from the surface of the nucleosomes, is composed of a variety of amino and is subject to extensive post-translational modifications that impacts transcriptional activation or inactivation, chromatin constitution and DNA replication (Iwasaki et al., 2013). Histone modifications include acetylation of lysine residues, methylation of lysine and arginine residues, ubiquitination of lysine residues, phosphorylation of serine and threonine residues, among others.

The acetylation and methylation of histones H3 and H4 are the most abundant post-translational histone modifications and, therefore, the most studied. Acetylation of lysine, for example, is associated with transcription status of active genes. On the other hand, the methylation of lysines and arginines, although generally associated with gene silencing, may also lead to the activation of gene transcription, depending on the region of insertion in the N-terminal histone and the number of methylations present (one, two or three) (Iwasaki et al., 2013).

There are two mechanisms through which histone acetylation regulates gene transcription. The first one is by reducing the positive charge of the histone proteins, thus decreasing their affinity to the DNA molecule (negatively charged). Consequently, there is a greater DNA exposure to transcription factors (Bannister and Kouzarides, 2011). The second is by creating, stabilizing or breaking regions of interaction between chromatin and regulatory proteins, such as transcription factors or proteins that act on chromatin condensation (Feinberg, 2001; Santos-Rosa and Caldas, 2005). Proteins with bromodomains, for example, are capable of recognizing the acetyl group in histones, making chromatin more accessible to remodelers and transcription factors (Kouzarides, 2007). The literature describes the lysines K5, K8, K12, K16 of histone H4 and the lysines K9, K14, K18, K23 of histone H3 as the best regions for the insertion of acetylation marks (Huynh et al., 2017).

Histone acetyl transferase (HATs) and histone deacetylase (HDACs) have been shown to regulate gene transcription by promoting the addition or removal, respectively, of the acetyl group to the N-terminal histone pool (Grunstein, 1997). There are 17 HAT enzymes, which are divided into two types, A and B. Type A HATs are found in the nucleus and regulate gene expression, working mainly as co-activators of transcription. Type B HATs are located in the cytoplasm, where they acetylate newly formed histones (Guo, 2009). The insertion of the acetyl group is done by specific HATs for each N-terminal region, i.e., the acetyl group present in acetyl-coenzyme A (Acetyl-CoA) is transferred to the ε-amino region of the target lysine (Galdieri et al., 2014). Removal of this group is done by the HDACs or KDACs, releasing acetate anions. There are 18 HDACs divided into class I (HDAC1-3 and 8), class II (HDAC4-7 and 9), class III or sirtuins (SIRT1-7), and class IV (HDAC11) (Vogelauer et al., 2012).

Another important post-translational modification of histones is the methylation of lysine and arginine amino acid residues. Most histone amino acid methylations are related to gene silencing. Unlike acetylation, histone methylation does not cause charge changes, but may cause conformational changes of the proteins, forming a specific binding site for other proteins (Bannister and Kouzarides, 2011).

The role of histone methylation in controlling gene expression depends on the location of the amino acid residue, where it is inserted, and the amount of methyl groups added. For example, the trimethylation of lysine 4 of histone 3 (H3K4me3) is associated with increased gene transcription and formation of euchromatin (decompressed form of chromatin). On the other hand, the triple methylation of lysines 9 or 27 in histone H3 (H3K9me3 and H3K27me3, respectively) is related to the reduction of transcription and the formation of heterochromatin (compact form of chromatin) (Park et al., 2007; Bártová et al., 2008). The presence of H3K9me3 can promote chromatin rearrangement. Heterochromatin 1 (HP1) protein binds to a histone methyltransferase (HMT) and promotes the trimethylation of other nearby H3K9. Consequently there is propagation of the heterochromatin structure along the chromosome until a delimiter is found (Stewart et al., 2005).

The enzymes responsible for the histone methylation are called histone methyltransferases (HMTs) and those responsible for the removal are called histone demethylases (HMDs). There are 3 classes of HMTs enzymes: lysine methyltransferases having SET domain, lysine methyltransferases lacking SET domain, and arginine methyltransferases (Teperino et al., 2010). The identification of the first histone lysine demethylase (KDM1A) is relatively recent (Shi et al., 2004). Other histone demethylase enzymes were then identified and are currently classified as belonging to the KDM1 family (KDM1A and KDM1B) and to the family of demethylases containing Jumonji C domain (JmJC - the largest group of histone demethylases) (D’Oto et al., 2016). The discovery of these enzymes demonstrates that histone methylations is a much more dynamic process than previously estimated, and the modifications can be inserted or removed according to the cell needs (Agger et al., 2008).

### Post-translational histone modification in bovine embryos

Post-translational modifications of histones play a crucial role in bovine embryonic development by allowing changes in the gene expression at specific times/regions. For example, soon after fertilization, in paternal origin chromosomes, the protamines are replaced by histones that present high levels of acetylation and low levels of methylation (Messerschmidt et al., 2014; Urrego et al., 2014). On the other hand, the maternal genome undergoes few changes, maintaining the same pattern of lysine acetylation and arginine methylation in H3 and H4 (Rodriguez-Osorio et al., 2011).

Soon after the first cleavages there is a reduction in the acetylation of lysine 9 and 18 in histone H3 (H3K9ac and H3K18ac, respectively); the lowest levels are observed in the 8-16 cells stage. After that, there is an increase in the acetylation of these lysines until the blastocyst stage when the acetylation levels of K18 are higher in trophectoderm cells than in the inner cell mass. Acetylation of lysines 5 and 8 of histone 4 (H4K5ac and H4K8ac, respectively), however, keep stable levels from the first cleavages up to the 8-16 cells stage, only showing an increase after this stage and with no difference between the acetylation profile between cells of the trophectoderm and the inner cell mass (Wu et al., 2011).

Regarding the presence of HATs and HDACs, there are few reports detailing the expression of these enzymes throughout bovine embryonic development. In one study, HDAC1, HDAC3, HDAC7, HAT1 and HAT2 mRNA have been observed in oocytes, 2-cell, 8-cell and blastocysts (McGraw et al., 2003). The same study observed that the expression of HAT1, but not of HAT2, differed throughout the development, being overexpressed at the blastocyst stage. Regarding the expression of HDAC1 and HDAC2, they were found to be increased in blastocysts when compared to 2 cells and 8-16 cells embryos. Meanwhile, HDAC3 and HDAC7 did not differ between the different stages of embryonic development (McGraw et al., 2003). These findings suggest that, in the blastocyst stage, simultaneous acetylation and deacetylation events are occurring, probably to ensure that these embryos are able to add and remove specific acetylation marks and activate gene expression as needed.

For histone methylation levels in bovine embryos, it has been reported that shortly after the first cleavages, there is a reduction in H3K9me3 until the 8-16 cells stage, while the dimethylation (H3K9me2) levels in this lysine remain constant. This mark is associated with Xist and long terminal repeats (LTRs) silencing in mouse embryos (Fukuda et al., 2014; Wang et al., 2018). After the 8-16 cell stage, there is an increase in di and trimethylations of this lysine up to the blastocyst stage (Fukuda et al., 2014; Wang et al., 2018). The methylation of H3K9 is particularly important because it appears to follow the same profile observed for DNA methylation, both epigenetic mechanisms capable of reducing gene transcription (Santos et al., 2003).

Other important histone repressive marks that have already been identified in bovine embryos are the trimethylation of lysine 27 and 64 of histone H3 and lysine 20 of histone H4 (H3K27me3, H3K64me3 and H4K20me3, respectively). Theses marks present the same reprogramming profile during the development, being remarkably reduced after fertilization and during the first cleavages, then increasing to result in blastocysts with abundance of these histone mark (Ross et al., 2008; Daujat et al., 2009; Wongtawan et al., 2011; Ross and Sampaio, 2018). Opposed to the other methylations previously described, the methylation of lysine 4 of histone H3 (H3K9me), a histone post-translational modification associated with increased gene transcription presents its higher levels at the beginning of development (first cleavages) and at later stages of development (blastocyst), showing its lower intensity in embryos around the 8-16 cells stage (Wu et al., 2011).

Among the enzymes that control histone methylation, some have already been studied in bovine embryos. SETDB1, a specific methyltransferase for H3K9, is abundant in oocytes and embryos up to the 8-cells stage, when it begins to decline and reaches the lowest levels in blastocysts (Golding et al., 2015). Meanwhile, SUV39H1 and SUV39H2, that also act on H3K9 methylation, exhibit high levels of expression only at the 2-cell and 4-cell stages, respectively (Zhang et al., 2012). However, SMYD3, an H3K4-specific methyltransferase is observed in lower levels throughout initial development until the morulae compactation, when a peak is observed in the amount of transcripts for this enzyme (Bai et al., 2016).

Regarding the enzymes that act in the histone demethylation processes, the presence of HDM3A, HDM4A and HDM4C during the embryonic development of bovine has been described, being observed in lower quantity only in embryos with 4 or 8 cells. HDM5B, another histone demethylase, is absent in the early stages of development, only being observed after the embryo reaches 8 cells (Sharp et al., 2018). Meanwhile, HDM4D and HDM4E (specific for H3K9me3 and H3K9me2), although detected at all stages of embryonic development, have increased expression in embryos at the 8-16 cells stage (Liu et al., 2018). JMJD3 presents high levels in oocytes, then it is reduced at the early embryo and increases again in blastocysts (Canovas et al., 2012). JMJD1C, another JmjC-domain-containing demethylase for H3K9me1 and H3K9me2 (Li et al., 2015), presents high levels throughout all the embryo development in bovine (Li et al., 2015).

The reports showing the association between post-translational histone modifications and the enzymes responsible for these modifications throughout bovine embryonic development show that the vast majority of these marks undergo drastic changes around the time of the major genome activation (approximately 8-16 cells). This demonstrates the intense reprogramming that these embryos are going through and suggests that this is a critical decision point that impacts the survival and correct development of the embryo.

### Covalent modifications in RNAs

Another well-known epigenetic phenomenon is the modification of N6-methyladenosine (m^6^A), a type of RNA methylation. Among more than 150 RNA modifications currently described, m^6^A is the most abundant in mammalian messenger RNAs, representing about 0.1–0.4% of adenosine residues in total cellular mRNA (Meyer et al., 2012). Although this modification has been known for some years, only recently its biological relevance began to be unveiled. Similarly to changes in DNA, the m^6^A is reversible and its presence is evolutionarily conserved in many species. This modification can modulate the flow of genetic information and the response to environmental challenges (Schaefer et al., 2017). Therefore, deciphering exactly how this modulation is done represents the breaking of a new frontier in reproductive biology. Overall, the m^6^A is found in long internal exons and is preferably enriched within 3 ’UTR regions, around stop codons. In addition to mRNAs, m^6^A is also present in long non-coding RNAs, such as XIST, small nuclear RNAs and ribosomal RNAs (Meyer et al., 2012).

This epigenetic mark is established by a protein complex called “writer” that includes three well-characterized components: METTL3, METTL14 and WTAP. The METTL3 acts as a binding subunit to the protein S-adenosyl methionine (SAM), while the METTL14 is responsible for the structural maintenance of the complex (Bokar et al., 1997). This complex preferably binds to RNA oligonucleotides containing the GGACU sequence (Harper et al., 1990). The reverse process of RNA demethylation is carried out by “erasers”, such as the enzymes FTO and ALKBH5.

The m^6^A is related to a series of metabolic processes that involve RNA such as the regulation of gene expression, mRNA stability, translation efficiency, alternative splicing and cytoplasmic turnover (Kasowitz et al., 2018). The functional roles of m^6^A are being gradually discovered mainly through experiments in which METTL3 was inactivated; such studies have shown that the loss of m^6^A compromises the fate of stem cells and pluripotency (Batista et al., 2014; Geula et al., 2015). In addition, this modification has also been described as being important in the extensive regulation of gene expression for fertility and development (Meyer and Jaffrey, 2014), such as observed for MAT2A mRNA methylation (SAM production) to mediate the downregulation of this mRNA under high-SAM conditions, in mouse embryos (Mendel et al., 2018). The functions of the m^6^A are mediated by a group of proteins of the YTH family (YTHDF1, YTHDF2, YTHDF3 and YTHDC1) that specifically recognize the methylated adenosines in the RNA. These binding proteins are called “m^6^A readers”. So, while methyltransferases (writers) and demethylases (erasers) establish a complex mechanism for regulating the location of the changes along the RNA, the readers mediate their biological functions. Thus, post-transcriptional RNA modifications allow for additional control of gene expression, serving as a powerful mechanism that directs the fate of groups of transcripts to be processed, exported to the cytoplasm, translated and degraded, eventually affecting protein synthesis (Hsu et al., 2017).

As far as we know, all data available in literature for epigenetic modification for bovine embryos were generated using *in vitro* produced embryos. The literature lacks reports about the reprogramming profile of *in vivo* produced embryos, considered the gold standard embryos, along the development. Also, it has been already reported that even slight changes in the conditions of *in vitro* can lead to alteration in molecular control (Leite et al., 2017). Taken together, that information demonstrates the importance of more studies to elucidate the changes occurring during the initial embryonic development in bovine embryos and, ultimately, what can be changed in in vitro conditions to guarantee an accurate epigenetic reprogramming.

### Metaboloepigenetics – more than meets the eyes

The concept of the crosstalk between metabolism and molecular control of cells is not recent. For more than two decades, there were reports associating changes in cell culture system with altered metabolism and gene expression profile. However, the mechanisms involved in this response began to be elucidated in other cell types only a few years ago.

**Figure 3:**
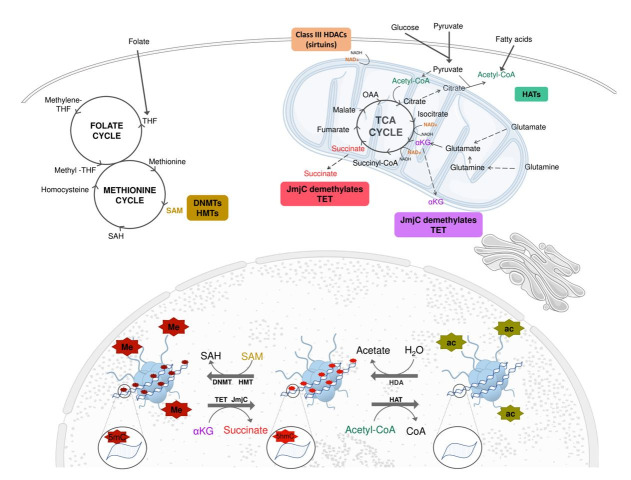
The folate cycle starts with the production of THF (tetrahydrofolate) from folate. THF is then converted into Methylene-THF and Methyl-THF, which is required for the production of methionine. The methionine can be converted to S-Adenosylmethionine (SAM) that acts as a methyl-group donor both DNA and histone methylation through the respective enzymes (DNMTs and HMTs). The consumption of the methyl group from SAM results in SAH (S-Adenosylhomocysteine), which can be converted to homocysteine and, again, to methionine. Glucose and pyruvate can produce acetyl-CoA, either by entering the mitochondria and being converted to citrate, or directly in the cytoplasm. Another source of Acetyl-CoA is the ß-oxidation of fatty acids. The acetyl-CoA generated can be used as an acetyl group-donor for histone acetylation by HATs (histone acetyl transferase enzymes). The removal of histone acetylation is promoted by HDAs enzymes and results in acetate production. Following in the tricarboxylic acid (TCA) cycle, the  α-Ketoglutarate (α-KG) can be produced and transported to the cytosol and, later be used as substrate by TET or JmjC domains containing enzymes that promote, respectively, DNA and histone demethylation. Another source of α-KG are glutamine and glutamate. The α-KG that remains in the TCA cycle is converted to succinate. Succinate might continue in TCA cycle by and ultimately lead to the production of oxaloacetate. However, the succinate can leave the mitochondria and prevent DNA and histone demethylations by blocking TET or JmjC domain containing enzymes activity, respectively. At different steps of the tricarboxylic acid (TCA) cycle the consumption of NAD^+^ is necessary. NAD^+^ modulate sirtuin activity, a specific group of HAT. DNMT-DNA methyltransferase; HMT-histone methyltransferase;  TET-ten-eleven translocation methylcytosine dioxygenase; HDAC-histone deacetylase, HAT-histone acetyltransferase. Adapted from SMART Servier Medical Art image bank (SMART, 2020).

Cellular metabolites have been described as enzyme co-factors, responsible for epigenetic changes or even as substrates for chemical modifications in nucleic acids or structural chromatin proteins, once again reinforcing the relationship between metabolism and the molecular profile of the cells (Figure 3). The characterization of these phenomena in embryos is even more recent (Ispada et al., 2018, 2020; Zhang et al., 2019b). Some of these mechanisms are better described below.

### Metabolic regulation of Histone acetylation and deacetylation

Acetyl-CoA is a central metabolic intermediate, which is generated in mitochondria as a byproduct of both glucose and lipid metabolism (β-oxidation). In mammalian cells, most cytosolic acetyl-CoA comes from citrate exported from mitochondria by an ATP-citrate lyase (ACL) catalyzed reaction. Increased glycolysis boosts the synthesis of citrate and thus the generation of cytosolic acetyl-CoA. Most of the acetyl group present in the histones is derived from the acetyl-CoA generated in this process (Fan et al., 2015).

The levels of intracellular acetyl-coA are influenced by several factors, such as oxygen tension and energy substrate availability. High glucose content in culture media of tumor cells, for example, leads to increasing in acetyl-CoA, with consequences in histone acetylation and gene expression (Lee et al., 2014). Inversely, quiescent fibroblasts present lower acetyl transfer to histones when compared to proliferating ones (Evertts et al., 2013). This ability of cells to respond to environmental conditions by changing the molecular framework is particularly important for appropriate decision making as cell survival, proliferation or differentiation, according to the metabolic status (Wellen and Thompson, 2012). Despite the fact that acetyl-CoA level is determinant for histone acetylation, it still remains controversial if this is a specific or a global response. The decrease of histone acetyltransferase activity as a consequence of mtDNA depletion interfere in histone acetylation of locus-specific gene expression that respond to this phenotype (Lozoya et al., 2019). However, decreased glucose availability in tumor cell lines diminished acetyl-CoA levels and histone acetylation in a global manner (Lee et al., 2014).

Histone acetylation is regulated by a balance between the activities of HATs and HDACs. In this sense, histone deacetylation, mediated by histone deacetylases (HDACs), is also a crucial event to transcriptional control and cell differentiation. Sirtuins belongs to Class III HDACs and are proteins with different cell functions, including metabolic regulation and deacetylation of histones at specific regions, as gene promoters, and thus controlling several specific cellular events. These class of enzymes respond to the levels of cellular NAD^+^, been related to the metabolic and redox status of the cell since it links TCA cycle and oxidative phosphorylation in mitochondria. In this sense, the deacetylase activity of sirtuins are regulated, at least in part, at three different levels: NAD^+^ biosynthesis, modulation of sirtuin activity by NAD^+^ and competitive use of NAD^+^ by other cellular processes (Imai and Guarente, 2016). Other small molecules may also regulate sirtuin activity, such as some specific fatty acids. In muscle cells, oleic acid stimulates the phosphorylation of SIRT1 Ser-434, increasing its catalytic deacetylase activity with consequences in fatty acid oxidation (Lim et al., 2013).

Distinct from class III, classes I and II HDACs are NAD^+^-independent but also affected by cellular metabolism. The administration of exogenous ketone body β-hydroxybutyrate to mouse lead to increased global histone acetylation in several organs, but mostly the kidneys, due to HDAC inhibition (Shimazu et al., 2013). The same effect was observed after somatic cell nuclear transfer zygotes exposure to β-hydroxybutyrate, in which the hyperacetylation state of H3K9 remained until blastocyst stage (Sangalli et al., 2018).

The significance of metabolic changes in acetylation patterns is better described in embryonic stem cells than gametes and embryos. In mouse ESC the glycolytic acetyl-CoA production promotes histone acetylation maintaining the pluripotency state. The modulation of glycolysis-derived acetyl-CoA leads to changes in histone acetylation and deacetylation levels, impacting cell differentiation (Moussaieff et al., 2015). The same pattern was recently evidenced in bovine embryos (Ispada et al., 2020). In this case, changes in histone acetylation levels were related to differences induced by pharmacological modulation of pyruvate metabolism in a dose-dependent manner. In the same work, the authors discuss that non-induced differences in blastocyst metabolism also lead histone acetylation changes probably by the translocation of PDC complex to the nucleus promoted by cellular stress conditions (Ispada et al., 2020; Zhou et al., 2020).

### Metabolic regulation of DNA/histone methylation and demethylation

Several chromatin-modifying enzymes, such as the DNMTs, TETs and histone demethylases use metabolic intermediates as cofactors or inhibitors, demonstrating a direct interaction between epigenetic regulation and metabolism. A good example of such interaction is SAM, a product of the one-carbon (1C) metabolism, that releases the methyl-group used by DNMTs enzymes to promote DNA methylation. Some nutrients are used as a substrate for one-carbon metabolic pathway, making methylation highly dependent on their availability. Methionine, for example, is the precursor of SAM and a key nutritional factor limiting its synthesis (Serefidou et al., 2019). Thus, fluctuations of methionine can influence DNA methylation and gene expression. Besides, as the establishment of epigenome is particularly vulnerable to metabolic dysfunction, especially during the prenatal stages, the maternal supplementation with methionine and folate can prevent abnormal fetus development and neural tubal defects (Kalhan, 2016). Alcohol ingestion during pregnancy also affect 1C cycle, leading to changes in DNA methylation pattern and teratogenic effects (Sharp et al., 2018).

Still in this context, the factors involved in one carbon metabolism also act as critical cofactors or inhibitors of histone modifiers, making the histone methylation status also tightly connected to the cells’ metabolic state (Serefidou et al., 2019). Recent studies demonstrated that the reduction of SAM induced by defects in methionine metabolism alter the dynamics of histone methylation. It was observed that the depletion of methionine in mouse ESCs leads to a decrease in H3K4 markers and reduces the expression of the pluripotency factor *NANOG*, inducing a more differentiated state (Tran et al., 2019b). Besides, in mice, a restriction in methionine intake rapidly triggered a decrease in H3K4 methylation modulated by SAM, thus reinforcing the sensitive relationship between intracellular levels of SAM and the activity of histone methylase enzymes (Mentch et al., 2015).

Another strong example that highlights the metabolic regulation of epigenetic mechanisms is α-ketoglutarate (αKG), that acts as a limiting factor in the tricarboxylic acid (TCA) cycle (Wu and Zhang, 2014; Tran et al., 2019a). This metabolite can be generated by glutamate deamination by the enzyme glutamate dehydrogenase or, in the TCA cycle, by decarboxylation of the isocitrate by the enzyme isocitrate dehydrogenase. In the TCA cycle, α-ketoglutarate is decarboxylated to succinyl-CoA and CO_2_ by α-ketoglutarate dehydrogenase, which in turn is converted to succinate (Berg et al., 2002).

Multiple studies demonstrated that the αKG: succinate ratio can affect the pluripotency status in both murine and human embryonic stem cells (Carey et al., 2015; TeSlaa et al., 2016). The accumulation of succinate and fumarate inhibits TET protein enzymatic activity, leading to higher levels of DNA methylation and consequently, the maintenance of a more differentiated state. On the contrary, pluripotent cells have high αKG: succinate ratio, higher TETs activity and reduction of DNA methylation (Carey et al., 2015).

Other reports also demonstrate that not only diet, but the *in vitro* environment affects DNA methylation levels (Urrego et al., 2014; Salilew-Wondim et al., 2015). In this sense, our group showed that alterations in the α-ketoglutarate: succinate ratio or their precursors in culture media influences DNA methylation in bovine embryos and they fail to perform DNA demethylation during the early stages of development (Ispada et al., 2018). Therefore, reprogramming leading to aberrant patterns of gene expression that could affect embryo viability and possibly offspring phenotype.

In a similar process as DNA, histone demethylases enzymes also use αKG as the cofactor to remove methyl groups on histones and to release succinate and formaldehyde. While αKG is crucial for histone demethylation, it was demonstrated that accumulation of succinate inside the cell can antagonize the activity of the histone demethylases and promote cellular differentiation in mouse and human embryonic stem cells (Carey et al., 2015; TeSlaa et al., 2016).

## Final remarks

For many years, embryo production systems were primarily aimed at increasing blastocyst rates and pregnancy, with most of the changes being suggested and implemented empirically. Advances in omics technologies associated with the solid development of bioinformatics tools represent a new era of possibilities for personalized culture systems targeting the breed, age of the donors, semen quality among other specific aspects. These interventions might not only increase quality and viability of embryos, but also help avoiding medium- and long-term consequences to the offspring.

**Figure 4:**
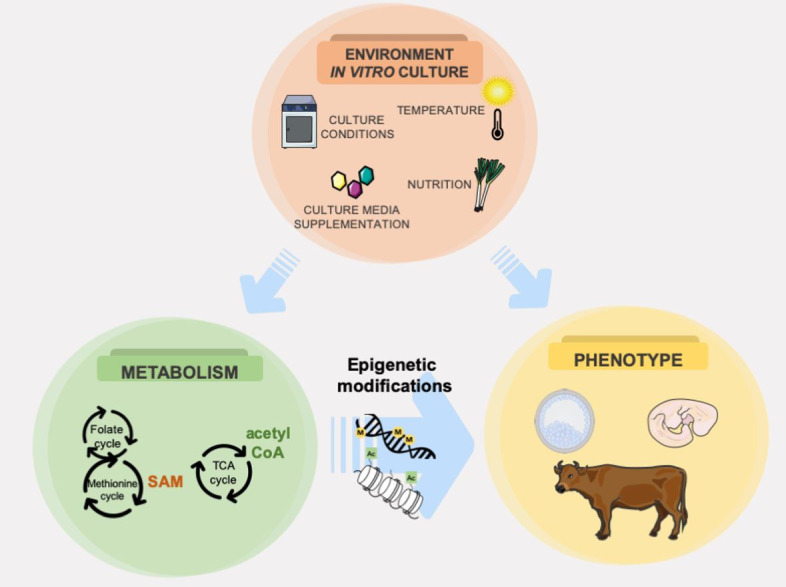
*In vivo* and *in vitro* environmental conditions are capable of influencing the phenotype both directly and indirectly. Indirectly, the environment may lead to changes in cell metabolism and affect the production of co-factors for epigenetic modifications. In this sense, the modifications of culture and production systems based on metabolic characteristics is a promising tool to improve embryo viability and animal production. SAM-S-adenosylmethionine. Adapted from SMART Servier Medical Art image bank (SMART, 2020).

The culture system is capable of imprinting marks on the embryos that remain throughout the development, causing consequences in their adult life and possibly to their future generation (Li et al., 2015). A classic example is the large offspring syndrome (LOS) that has been linked to the use of serum during embryo culture *in vitro*. This syndrome is characterized in cattle as derived from the misregulation of non-imprinted genes and loss-of-imprinting in specific genes during the early stages of development as consequence of non-optimized ART conditions (Chen et al., 2017). Despite that, it opens the possibility of inserting positive signatures in animals epigenomic when designing new culture media and promoting changes to the in vitro culture system (Figure 4).

To achieve this goal, metabolic and environmental conditions can be manipulated both in animal production and *in vitro*. Manipulation of the food intake and thermal control are some parameters that can improve the quality of gametes *in vivo*. *In vitro*, distinct supplementation (as the amount of amino acids, metabolites and lipids), physical conditions (as oxygen tension, culture media viscosity) allows the modification of morphophysiological parameters of the cells, as well as epigenomic marks in the nucleus (Leite et al., 2017; Ispada et al., 2020). Furthermore, mitochondrial DNA methylation, recently described in oocytes and embryos may also be a promising target for increasing embryo viability and animal production (Sirard, 2019).

In terms of embryo selection, metabolomics is proven to better predict quality than genomic marks (as SNPs), since metabolites are the result of genome/epigenome and environment interactions (Nicholson and Lindon, 2008; dos Santos et al., 2016). In this sense, mastering the metabolic regulation of epigenetic events is not only an intellectual pursuit, but also a powerful tool to drive specific changes on *in vitro* culture systems to generate more viable embryos and a healthier offspring.
